# An ecological cost associated with protective symbionts of aphids

**DOI:** 10.1002/ece3.991

**Published:** 2014-02-20

**Authors:** Sarah Polin, Jean-Christophe Simon, Yannick Outreman

**Affiliations:** 1 UMR 1349 IGEPP, AGROCAMPUS OUESTF-35042, Rennes, France; 2 Université EuropéenneBretagne, France; 3 UMR 1349 IGEPP, INRAF-35653, Le Rheu, France

**Keywords:** Behavioral divergence, *Hamiltonella defensa*, ladybird, parasitism, pea aphid, predation rate, protective symbiosis

## Abstract

Beneficial symbioses are widespread and diverse in the functions they provide to the host ranging from nutrition to protection. However, these partnerships with symbionts can be costly for the host. Such costs, so called “direct costs”, arise from a trade-off between allocating resources to symbiosis and other functions such as reproduction or growth. Ecological costs may also exist when symbiosis negatively affects the interactions between the host and other organisms in the environment. Although ecological costs can deeply impact the evolution of symbiosis, they have received little attention. The pea aphid *Acyrthosiphon pisum* benefits a strong protection against its main parasitoids from protective bacterial symbionts. The ecological cost of symbiont-mediated resistance to parasitism in aphids was here investigated by analyzing aphid behavior in the presence of predatory ladybirds. We showed that aphids harboring protective symbionts expressed less defensive behaviors, thus suffering a higher predation than symbiont-free aphids. Consequently, our study indicates that this underlined ecological cost may affect both the coevolutionary processes between symbiotic partners and the prevalence of such beneficial bacterial symbionts in host natural populations.

## Introduction

Within antagonistic interactions, interest divergence leads to the famous perpetual arms race between victims and enemies. The victims establish diverse defensive strategies that are selected for the benefits they provide in case of enemy attack. The evolution of defensive strategies also implies, however, that there are associated costs. The fitness costs of defense can arise from both internal processes, when fitness-limiting resources are allocated to defenses at the expense of growth and reproduction, and external processes (i.e., ecological costs) that occur when defense expression results in disrupting a beneficial interaction or in enhancing susceptibility toward enemies. Although the cost-benefit paradigm motivated most of the theory about the evolution of defense strategies against enemies (e.g., Strauss et al. 2002 on plants; Kraaijeveld et al. 2002 on animals), demonstrating the existence of fitness costs associated with defenses is a challenging task.

Most insects suffer attack from a range of natural enemies, including predators, parasites, pathogens and parasitoids. Because microorganisms, especially bacteria, have the capacity to make a diverse assortment of biologically active compounds, including toxins, insects could benefit from microorganism partners capable of providing additional protection against enemies. Recent work on diverse invertebrates has revealed that heritable facultative symbionts can confer protection against a range of natural enemies (Oliver et al. 2010). Apart from their essential nutrient-providing symbiont *Buchnera aphidicola*, many aphid species also harbor several facultative bacterial symbionts influencing various aspects of host ecology. In the pea aphid *Acyrthosiphon pisum* (Harris), resistance against parasitoids is provided by the infection with the *γ*-proteobacterium, *Hamiltonella defensa* (Oliver et al. 2003). In nature, *H. defensa* is commonly associated with the Pea Aphid X-type Symbiont (called PAXS); higher parasitism resistance under fluctuating temperature conditions is observed when this double infection occurs (Guay et al. 2009).

The *H. defensa* infection can induce, however, detrimental effects on host fitness by reducing its survival and reproductive success, suggesting a protective symbiosis cost (Oliver et al. 2006; Simon et al. 2011; Vorburger and Gouskov 2011). Moreover, in presence of parasitoids, aphids infected with *H. defensa* exhibit less defensive behaviors than uninfected ones (Dion et al. 2011). The reduction in defensive behavior – a general response to enemies' attacks – in infected aphids would then increase their susceptibility against predators. To test this possible ecological cost of protective symbiosis, we compared the predation susceptibility, induced by behavioral defense investment, of *A. pisum* individuals infected or not by protective symbionts.

## Materials and Methods

### Aphids and predator ladybirds

Eleven aphid clones were selected, according to their symbiotic status among a collection of natural clones maintained in the laboratory. A choice was made between natural aphid clones and aphid clones artificially manipulated for their symbiotic composition. In aphids, facultative symbionts can be manipulated by selective elimination with antibiotics and transfection from infected to uninfected aphids. This procedure allows the use of identical genetic aphid backgrounds with different symbiotic status. However, these elimination/transfection manipulations may induce many artifactual and detrimental effects. Moreover, this artificial symbiosis may produce some invalid partnerships due to incompatibilities between the symbiont genotype and the host genotype. Furthermore, behavioral divergence between symbiotic and aposymbiotic aphids facing parasitoids is large enough to be detected even by considering different genotypes (Dion et al. 2011); the same pattern was expected for predation avoidance. Consequently, we preferred not using aphids manipulated for their symbiont composition but aphid clones originated from individuals sampled in nature. In *A. pisum*, facultative symbiont infections are correlated with other aphid ecological traits such as body color and host–plant preferences (Frantz et al. 2009), and therefore, all the selected aphid clones were green and originated from alfalfa (*Medicago sativa*) fields where the prevalence of *H. defensa* in aphid populations is high (Frantz et al. 2009). As the co-occurrence of *H. defensa* and PAXS is common in natural populations (Guay et al. 2009; Henry et al. 2013), this association was considered further. The eleven clones were also all genetically distinct: this was assessed by genotyping several polymorphic microsatellites and removing clonal copies from the collection (Peccoud et al. 2009). The presence of the bacterial symbionts (*Hamiltonella defensa*,*Regiella insecticola*,*Rickettsia* sp., *Spiroplasma* sp., *Serratia symbiotica*, PAXS, and Rickettsiella) was detected as described in Peccoud et al. (2013). Finally, the selected clones included four clones free of any of the facultative symbionts known in *A. pisum*, three clones harboring *H. defensa* singly and four clones infested with both *H. defensa* and PAXS – mentioned hereafter as doubly infected clones.

*Adalia bipunctata* (Linnaeus), the two-spotted ladybird, a generalist predator of aphids, was provided by a commercial supplier (Koppert®, Berkel en Rodenrijs, The Netherlands) and maintained on various *A. pisum* clones in the laboratory for at least three generations prior to experiments. Ladybirds used for experiments were reared individually until their third molt was recorded. Fourth instar larvae were then starved for 24 h prior to the experiment to obtain predators of similar age and nutritional status.

All insects were maintained on broad bean *Vicia faba* (Linnaeus) under a long day regime (16 h of light) in climate rooms (20°C, 70% ± 10% relative humidity).

### Experimental setup

The foraging behavior of *A. bipunctata* and the behavioral responses of *A. pisum* were observed in a glass cage (18 × 22 × 25 cm) containing one *A. pisum*-infested broad bean leaf. This excised leaf was maintained horizontally within the middle of the cage. Ten aphids of the same clone were tested together as they reached their third larval instar. One fourth instar ladybird was placed on the leaf 1 h after aphids established. Behavior observation and recording then began using the event recorder “The Observer” (Noldus Information Technology©, Wageningen, The Netherlands) adjusted with a 0.1 sec resolution. Foraging behavior of the predator was recorded as series of searching and resting times (for honeydew feeding, remaining still or grooming after feeding) punctuated by encounters with aphids. Mobility was regarded as search activity. Attacks occurred when contact between an aphid and the predator was established physically. The outcome of an attack depended on whether the aphid was consumed or not. Two aphid defensive behaviors leading to attack failure were recorded: aggressiveness (i.e., quick motions of legs and/or body repelling the predator to undertake an attack) and evasiveness (walking or dropping away). Escape behavior may also be affected by just the presence of the predator alone aphids becoming aware of the presence of the predator, without any direct contact with the predator. Whatever their circumstances, evasion events were noted. Observation ended when either the ladybird or all the aphids left the leaf, or after 1 h of continuous observation. Pea aphid lines were assayed in randomized order to limit the influence of temporal variation in insect physiological status (aphids and ladybirds) on the outcome for each aphid line. The proportion of predation (the number of predated aphids over the 10 aphids initially exposed to predation) was then calculated. This procedure was repeated 112 times (about ten replicates per aphid clone).

### Data analyses

Three symbiotic statuses were considered: aphids were either uninfected, singly or doubly infected. The effect of the symbiotic status was tested on the following recorded dependent variables: the total number of ladybird attacks, the proportion of unsuccessful attacks due to aphid defensive behavior (aggressive or escape reaction), the overall number of aphid evasive events and the final predation proportion. In experiments, several clones per symbiotic status were tested. In the statistical modeling, we considered the symbiotic status of aphids as a fixed factor and the clone tested within a symbiotic status level as a random factor (i.e., the clone factor was nested in the symbiotic status one). This random factor extracts the aphid genotype's influence on dependent variables and strongly reduces the confounding effect between symbiotic status and aphid genotype. Therefore, to test whether the dependent variables differed among the different symbiotic status of aphids, we fitted generalized linear mixed models using *lme4* (Bates et al. 2009) in R 3.0.2 (R Development Core Team 2013) assuming either a Poisson (for count data) or binomial (for proportions) error and a log-or logit-link function, respectively. To assess the significance of the individual model terms, we used likelihood ratio tests.

## Results

**Figure 1:**
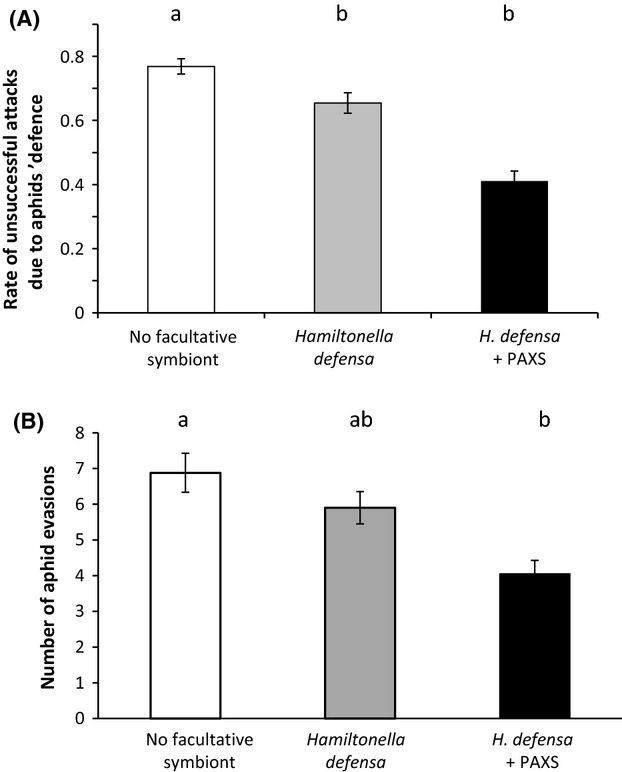
Effect of infection by *Hamiltonella defensa* alone or in association with Pea Aphid X-type Symbiont (PAXS) on the behaviors of *Acyrthosiphon pisum* aphids measured in (A) the proportion of *Adalia bipunctata* attacks that aborted due to *A. pisum* aphids' behavioral defense; and (B) the number of *A. pisum* individuals moving away during the *A. bipunctata* predatory search and attacks. White, gray and black bars represent colonies of aphids uninfected, and infected either with *H. defensa* alone or in association with PAXS, respectively. Statistical significance was evaluated with GLMM procedures and differences indicated by distinct letters above means and standard error.

No significant difference was observed in the total number of ladybird attacks according to the symbiotic status of the exposed aphids (about 7 attacks per experiment; Table 1). The proportion of unsuccessful attacks due to aphids' defenses was higher for uninfected aphids compared with aphids harboring at least one protective symbiont (Fig. 1A, Table 1). No difference was found between singly and doubly infected aphids. The overall number of aphid evasive behaviors, induced by the predator attack or its presence in the colony, depended on the symbiotic status of aphids (Fig. 1B, Table 1): doubly infested aphids were less likely to evade than uninfected aphids and aphids harboring *H. defensa* singly presented an intermediate number of evasion events.

**Table 1:** Results from generalized linear mixed model (GLMM) analyses. Estimates are either the model coefficients associated with each level of the fixed factor or the variance associated with the random factor.

	Number of ladybird attacks		Proportion of unsuccessful attacks due to aphids' defense		Number of aphid evasions		Proportion of predation in aphid colony	
	Estimates	Test statistics	Estimates	Test statistics	Estimates	Test statistics	Estimates	Test statistics
Aphid symbiotic status (fixed factor)								
No protective symbionts	0.000	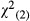 = 1.15^NS^	0.000	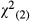 = 7.49*	0.000	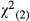 = 6.86*	0.000	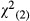 = 6.39*
*Hamiltonella defensa*	0.002^NS^		−0.742*		−0.177^NS^		0.711**	
*H. defensa* + Pea Aphid X-type Symbiont	−0.135^NS^		−1.279***		−0.542***		0.929**	
Aphid genotype (random factor)	0.026	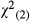 = 10.50 **	0.104	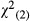 = 5.46^NS^	0.027	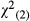 = 5.48^NS^	0.109	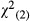 = 8.87*

NS *P* > 0.05;

* *P* < 0.05;

** *P* < 0.01;

*** *P* < 0.001.

**Figure 2:**
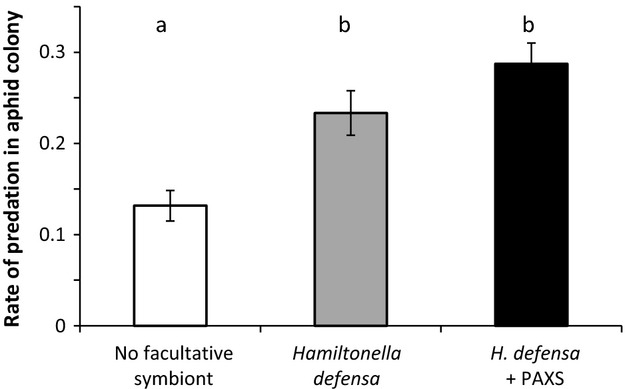
The proportion of aphids consumed by *Adalia bipunctata* on aphid colonies. White, gray and black bars represent colonies of aphids uninfected and infected either with *Hamiltonella defensa* alone or in association with Pea Aphid X-type Symbiont, respectively. Statistical significance was evaluated with a GLMM procedure and differences indicated by distinct letters above means and standard errors.

The resulting proportion of predation was twice lower for aphids free of facultative symbionts than for infected ones (Fig. 2, Table 1), without significant difference between singly and doubly infected individuals.

## Discussion

Our results indicate that aphids hosting *H. defensa* alone or in association with PAXS suffer higher predation than uninfected ones. This higher predation proportion results from behavioral variation among aphid clones with different symbiont component, infected aphids exhibiting less aggressive and evasive behavior in presence of predators. By enhancing susceptibility toward predation, our experiments suggest that protective symbioses result in associated ecological costs. Resistance to an aphid's enemy has already been hypothesized to be traded off by resistance to others (e.g., between different parasitoid species and between parasitoids and pathogenic fungus); but data in favor of this hypothesis are lacking (Ferrari et al. 2001). Conversely, the present study shows that symbiont-mediated resistance against parasitoids enhances susceptibility toward predators.

Protective symbioses in aphids have been identified here as influencing behavioral variation in host populations. This symbiont-mediated change in defensive behaviors in *A. pisum* has been also showed for aphid natural clones exposed to parasitoids (Dion et al. 2011). Such symbiont-mediated phenotype would be then no enemy specific: whatever the natural enemy foraging an aphid colony, individuals infected with *H. defensa* or doubly infected with both *H. defensa* and PAXS would exhibit less defensive behaviors compared with uninfected ones. The factor responsible for this behavioral variation is unknown. Individuals harboring *H. defensa* suffer from reduced survival and reproductive success (Oliver et al. 2006; Simon et al. 2011 in *A. pisum* hosts; Vorburger and Gouskov 2011 in *Aphis fabae* hosts), the reduced defensive behaviors could be an additional detrimental consequence of symbiont infection.

Most insect organisms are host to a diverse community of facultative symbionts. In natural populations of *A. pisum*,*H. defensa* is commonly associated with PAXS, this symbiotic association enhancing the parasitism resistance under variable temperature conditions (Guay et al. 2009). A host individual represents a habitat with limited space and energy, and this may lead to competition between symbionts and higher fitness costs for multiply infected hosts (Oliver et al. 2006). Even if the symbiont-mediated behavioral variation is more pronounced in doubly infected aphids (Fig. 1), multiple infections in *A. pisum* have no effect on the magnitude of the ecological costs (Fig. 2).

In nature, aphids are resources for a great number of natural enemies, including parasitoids and predators. The ecological costs associated with the infection of protective symbionts, added to the direct fitness costs, raise questions about the maintenance of these symbionts in natural populations. The prevalence of *H. defensa* highly varies among natural aphid populations and is particularly high in populations specialized on alfalfa (*Medicago sativa*; Frantz et al. 2009). The persistence of *H. defensa* in host populations may depend on (1) the balance between the benefits provided to the host and the associated costs of symbiosis; (2) the transmission rate of the protective symbiont within the aphid populations; and (3) the ecological context (see Kwiatkowski and Vorburger (2012) for theoretical evidence). By affecting aphid's ecology through both protection against parasitoids and higher susceptibility to predation, the evolutionary relationship between host and protective symbionts may indeed depend on the interplay between predation and parasitism pressures and then be selectively favored in some environments, but not in others. Further empirical and field works are thus needed to assess the effects of spatial and temporal dynamics in enemy pressures on the persistence of beneficial symbioses in natural host populations.

Symbiotic relationships have traditionally been classified as beneficial, detrimental or neutral depending on the effects on partners. Time is ripe for a new vision of symbiosis: an inclusive understanding of both symbiont-driven evolution and maintenance of symbioses in host populations requires a consideration of the diversity of effects on partners, combining beneficial and detrimental effects. However, demonstrating the existence of indirect detrimental effects like ecological costs may be challenging. The detection of ecological costs requires an intimate knowledge of the ecological web including the target species. Given the complexity of ecological webs, it is likely that many ecological costs associated with symbiosis remain to be discovered.
